# Probiotic Potential Analysis and Safety Evaluation of *Enterococcus durans* A8-1 Isolated From a Healthy Chinese Infant

**DOI:** 10.3389/fmicb.2021.799173

**Published:** 2021-12-14

**Authors:** Yi Zhou, Lu Shi, Juan Wang, Jia Yuan, Jin Liu, Lijuan Liu, Rong Da, Yue Cheng, Bei Han

**Affiliations:** ^1^School of Public Health, Health Science Center, Xi’an Jiaotong University, Xi’an, China; ^2^Department of Clinical Laboratory, The First Affiliated Hospital of Xi’an Jiaotong University, Xi’an, China; ^3^Key Laboratory for Disease Prevention and Control and Health Promotion of Shaanxi Province, Xi’an, China

**Keywords:** *Enterococcus durans*, stress tolerance, probiotic characters, safety evaluation, whole-genome sequencing

## Abstract

To evaluate the probiotic characteristics and safety of *Enterococcus durans* isolate A8-1 from a fecal sample of a healthy Chinese infant, we determined the tolerance to low pH, survival in bile salts and NaCl, adhesion ability, biofilm formation, antimicrobial activity, toxin gene distribution, hemolysis, gelatinase activity, antibiotic resistance, and virulence to *Galleria mellonella* and interpreted the characters by genome resequencing. Phenotypically, *E. durans* A8-1 survived at pH 5.0 in 7.0% NaCl and 3% bile salt under aerobic and anaerobic condition. The bacterium had higher adhesion ability toward mucin, collagen, and Bovine Serum Albumin (BSA) *in vitro* and showed high hydrophobicity (79.2% in chloroform, 49.2% in xylene), auto-aggregation activity (51.7%), and could co-aggregate (66.2%) with *Salmonella typhimurium*. It had adhesion capability to intestinal epithelial Caco-2 cells (38.74%) with moderate biofilm production and antimicrobial activity against several Gram-positive pathogenic bacteria. A8-1 can antagonize the adhesion of *S. typhimurium* ATCC14028 on Caco-2 cells to protect the integrity of the cell membrane by detection of lactate dehydrogenase (LDH) and AKP activities. A8-1 also helps the cell relieve the inflammation induced by lipopolysaccharide by reducing the expression of cytokine IL-8 (*P* = 0.002) and TNF-α (*P* > 0.05), and increasing the IL-10 (*P* < 0.001). For the safety evaluation, A8-1 showed no hemolytic activity, no gelatinase activity, and had only *asa1* positive in the seven detected virulence genes in polymerase chain reaction (PCR), whereas it was not predicted in the genome sequence. It was susceptible to benzylpenicillin, ampicillin, ciprofloxacin, levofloxacin, moxifloxacin, tigecycline, nitrofurantoin, linezolid, vancomycin, erythromycin, and quinupristin/dalofopine except clindamycin, which was verified by the predicted *lasA*, *lmrB*, *lmrC*, and *lmrD* genes contributing to the clindamycin resistance. The virulence test of *G. mellonella* showed that it had toxicity lower than 10% at 1 × 10^7^ CFU. According to the results of these evaluated attributes, *E. durans* strain A8-1 could be a promising probiotic candidate for applications.

## Introduction

Gut microbiota contributes a lot to human health and the occurrence of diseases. It is called the “invisible endocrine organ,” which is the place where the body digests food and absorbs nutrients (Scarpellini et al., 2010). *Enterococcus*, as one of the indigenous bacteria in the intestine, belongs to the class of facultative anaerobic lactic acid bacteria (LAB). *Enterococcus* spp. is distributed widely and can be separated from the environment, food, and human and animal gastrointestinal tract and has strong resistance to harsh stress and can survive at different conditions (Starke et al., 2015; Cirrincione et al., 2019).

Probiotics are defined as “live microorganisms which consumes in sufficient amounts, affect beneficially the health of the host [*sic*].” Enterococci have biological properties of probiotics; some strains usually show high resistance to acids and bile salts (Gu et al., 2008), antioxidant and free radical scavenging activity, improve host immunity by intestinal adhesion and localization (Starke et al., 2015; Li et al., 2018), and enhance apoptosis of human cancer cells (Nami et al., 2014); at the same time, some strains have antibacterial activity and anti-inflammatory effects (Popović et al., 2019). With the continuous discovery and exploration of the probiotic characteristics of *Enterococcus*, many strains have proven to be effective and safe and developed into applications, such as the commercial microecological probiotics in human (Medilac-Vita^®^, live *Bacillus subtilis* and *Enterococcus faecium*) and veterinary medicine (Bonvital^®^, *E. faecium* DSM 7134) (Li et al., 2020), and the food industry (Cernivet^®^, *E. faecium* SF68^®^; Symbioflor^®^, *E. faecalis*) (Strompfová et al., 2004).

At the same time, *Enterococcus* has both probiotic character and potential pathogenicity, and some strains can cause important infections and diseases, such as endocarditis; bacteremia; and urinary, intra-abdominal, pelvic infections, and central nervous system infections (O’Driscoll and Crank, 2015). It is generally known that antibiotic resistance and virulence are the main factors for *enterococci* pathogenicity. The main concern for the safety evaluation of *enterococci* is focused on the potential infectivity and transferable drug-resistant genes (Yang et al., 2015). Pathogenic enterococci may cause concerns about the safety using of probiotics, so to screen the potential probiotic enterococci, assessing and evaluating the safety is necessary and a priority.

*Enterococcus* is one of the most controversial LAB (Martino et al., 2018). The development of new enterococcal probiotics needs a strict assessment with regard to safety aspects for selecting the truly harmless strains for safe applications. The potential probiotic enterococci isolates can be applied to biotechnology development, and a broader application can be obtained by strain improvement. The aim of this study was to evaluate the probiotic characteristics and safety of *E. durans* A8-1 isolated from a fecal sample of a healthy Chinese infant and its potential in future probiotic development and application.

## Materials and Methods

### Bacterial Strains and Cell Culture

*Enterococcus durans* A8-1 were isolated from a fecal sample by the microbiology lab of the Nutrition and Food Safety Engineering Research Center of Shaanxi province, Xi’an, China. Fecal samples were taken from a healthy infant born 1–7 days earlier at the Maternal and Child Health Hospital of Bin County of Shaanxi province, China. There was no history of being treated with antibiotics after birth. The fecal sample was collected after the informed consent form was signed by the guardian. This study was reviewed and approved by the ethics committee of the Health Science Center, Xi’an Jiaotong University, Xi’an, China (No. 2016114).

*Enterococcus durans* A8-1, *Enterococcus faecalis* ATCC29212, *Lactobacillus rhamnosus* GG BL379, and *Bifidobacterium infantis* CICC6069 were inoculated into de Man, Rogosa and Sharpe (MRS) medium (CM187, Beijing Land Bridge Technology Co., Ltd., Beijing, China) and incubated aerobically with constant temperature shaker at 37°C for 18–24 h. About the streak-plating growth, A8-1 and BL379 were cultured on MRS agar using MRS broth with 15 g/L agar for 18 h. For the anaerobic culture, the bacterial cells were inoculated on the same medium and incubated in an anaerobic chamber (Coy Laboratory Products Inc., Ann Arbor, MI, United States) with a modified atmosphere of 82% N_2_, 15% CO_2_, and 3% H_2_ without shaking. For the growth of *Staphylococcus aureus* ATCC25923, *Pseudomonas aeruginosa* PA01, *P. aeruginosa* ATCC27853, *Enterococcus hormaechei* ATCC700323, *Salmonella typhimurium* ATCC14028, and *Escherichia coli* ATCC35218, nutrient broth was used (CP142, Beijing Land Bridge Technology Co., Ltd., Beijing, China).

Caco-2 cells were cultured in Dulbecco’s modified Eagle’s minimal essential medium (DMEM, Hyclone) supplemented with 10% fetal bovine serum (FBS, Hyclone) without antibody. The cells were kept at 37°C in an atmosphere containing 5% CO_2_.

### Isolation and Identification of A8-1

One gram of stool sample was mixed with 0.9% sterile saline solution to a final volume of 10 mL, and 0.1 mL of this dilution was spread on the MRS agar plate (MRS broth with 15 g/L agar) and cultured anaerobically at 37°C for 48 h. After incubation, colonies were randomly selected from each sample and subcultured on MRS plates for further analysis. Single colonies were picked out for Gram staining and microscopic observation and catalase, oxidase production, and nitrate reduction tests (Mansour et al., 2014).

For further confirmation, the 16S rRNA gene sequence (1.4 kb) was amplified, and sequenced by Sangon Biotech (Shanghai, China) Co., Ltd. Primers used were 16S-27F: 5′ AGA GTT TGA TCC TGG CTC AG 3′, 16S-1492R: 5′ GGT ACC TTG TTA CGA CTT 3′. The polymerase chain reaction (PCR) amplification conditions were as follows: initial denaturation for 5 min at 94°C, 35 cycles of denaturation for 30 s at 94°C, annealing at 60°C for 30 s, extension at 72°C for 60 s, and a final elongation step of 5 min at 72°C. Multiple alignments with sequences of closest similarity were analyzed using CLUSTAL W, and a phylogenetic tree was constructed by using the neighbor-joining method.

### Acid, NaCl, and Bile Salt Tolerance

A8-1 was inoculated into MRS broth at 37°C overnight in an aerobic incubator and anaerobic chamber separately. The overnight culture was centrifuged, and the collected cells were washed twice by sterile phosphate buffered solution (PBS) and resuspended in OD_600_ = 0.1 at fresh MRS with different pH 3.0, 4.0, and 5.0; bile salts (0.5, 1, 2, and 3%) and NaCl (1.75, 3.5, and 7%). The negative control was MRS blank medium at pH 6.5. Three replicates were set for each medium. Growth was monitored by optical density at 600 nm every 30 min at 37°C for 21 h in a microtiter plate reader (PolarStar, BMG Labtech, Germany) (Banwo et al., 2013; Li et al., 2020). Maximal growth, the lag phase duration, and the increment in OD values were considered by using the Gompertz growth analysis mode of non-linear regression in GraphPad Prism 7 (Wang et al., 2020).

### Antibacterial Ability

The minimum inhibitory concentration (MIC) method was used to determine the antibacterial activity of A8-1. The indicator bacteria were as follows: *E. faecalis* ATCC29212, *S. aureus* ATCC25923, *P. aeruginosa* PA01, *P. aeruginosa* ATCC27853, *E. hormaechei* ATCC700323, *S. typhimurium* ATCC14028, and *E. coli* ATCC35218. A8-1 was inoculated into MRS broth and incubated at 37°C for 24 h. The cells were removed by centrifugation at 9,710 × *g* for 2 min at 4°C. The supernatants were filter sterilized and added to 96-well plates at 0, 25, 50, 100, 150, and 200 μL, made up to 200 μL with fresh medium. Finally, each test indicator bacteria was added to the well at the concentration of OD_600_ = 0.1. Growth of test indicator bacteria was monitored every 30 min by optical density at 600 nm with an automatic microplate reader for 12 h at 37°C (Wang et al., 2020).

### *In vitro* Hydrophobicity, Auto-Aggregation, and Co-aggregation

The hydrophobicity, auto-aggregation, and co-aggregation assays were performed according to Fonseca et al. (2021). The cell surface hydrophobicity of each strain was assessed by measuring microbial affinity to xylene and chloroform. The A8-1 was incubated overnight and washed in PBS twice, and then resuspended in PBS with OD_600_ of 0.8 (A0). Then, 1 mL xylene and 1 mL chloroform were added separately to 3 mL of A8-1 cell suspension and mixed thoroughly. Then, the water and xylene phases were separated for 30 min at room temperature. The aqueous phase was removed, and the new OD_600_ was measured (A1). The cell surface hydrophobicity (%) was calculated using the following formula: Hydrophobicity (%) = [(A0−A1)/A0] × 100%. The strain was classified into low (0–29%), moderate (30–59%), and high hydrophobicity (60–100%).

For auto-aggregation, A8-1 was incubated overnight and washed in PBS twice and then resuspended in PBS with OD_600_ about 0.6 (A0). Bacterial cell suspensions were vortexed for 10 s and subsequently incubated at room temperature for 5 h, and the new OD_600_ was measured (At). The auto-aggregation percentage was determined using the following equation:

Auto-aggregation(%)=(1-At/A0)×100%.

For co-aggregation, *E. durans* A8-1 and *S. typhimurium* ATCC14028 were incubated overnight separately and washed in PBS twice and then resuspended in PBS with OD_600_ about 0.8. Equal volumes (2 mL) of A8-1 and *S. typhimurium* ATCC14028 were mixed and incubated at room temperature without agitation for 5 h. Control tubes contained 2 mL of the suspension of each bacterial cells. The OD_600_ of the mixtures and controls were measured after incubation. The percentage of co-aggregation was calculated using the following formula: Co−aggregation (%) = [(Ax + Ay)/2−A(x + y)]/(Ax + Ay) × 100%, where Ax and Ay refer to the OD_600_ of the A8-1 and *S. typhimurium* ATCC14028 cell suspension, respectively, Ax + y represents the absorbance of the mixed bacterial suspension tested after 5 h.

### *In vitro* Binding to Bovine Serum Albumi, Mucin, and Collagen

Strain binding to different substrates was evaluated as reported previously (Muñoz-Provencio et al., 2009; Wang et al., 2020). Mucin (500 μg/mL, porcine stomach, Sigma-Aldrich, St. Louis, MO, United States), Bovine Serum Albumi (BSA) (500 μg/mL, Sigma-Aldrich, St. Louis, MO, United States), and collagen (50 μg/mL, type I, Roche, Mannheim, Germany) were added separately to the 96-well microplates and incubated overnight at 4°C. Then, wells were washed three times with PBS and dried at room temperature. Two milliliters of A8-1 were labeled by 20 μL cFDA [5-(6-)-carboxyfluorescein diacetate, Sigma Aldrich, St. Louis, MO, United States] to the plate wells. After mixing, the cells were incubated at room temperature for 1 h and kept away from light. The strain labeled by cFDA was added to the plate wells and separately incubated with immobilization at 4°C overnight and kept away from light. After incubation, each well was washed three times by PBS and dried at room temperature. The 100 μL cFDA-labeled A8-1 cells were added into wells with no immobilization and set as control. Fluorescence intensity of the well plate was measured by a microplate reader, and the adhesion rate of A8-1 cells to mucin, collagen, and BSA was calculated according to the following formula. *L. rhamnosus* GG BL379 was used as a positive control; its adhesion rate was set as 100%, and the relative adhesion of A8-1 cell to BL379 was calculated.

Adhesion (%) = (fluorescence intensity of A8-1)/(the free cFDA-labeled BL379) × 100.

### Adhesion Ability to Caco-2 Cells

The A8-1 was incubated overnight and washed in PBS twice and then resuspended in 1 mL DMEM medium (without antibiotic) with a final concentration of 10^7^ CFU/mL. Caco-2 cells cultured by high-glucose DMEM were seeded in 96-well plates and incubated at 37°C. The 200 μL of A8-1 suspension was added to each well containing Caco-2 cells and then incubated for 2 h. Caco-2 cells with DMEM was set as control. The Caco-2 cells were collected and washed three times by PBS to remove the unadhered A8-1. Then, trypsin was added to Caco-2 cells to lyse the adherent A8-1. Finally, the mixture of each well was cultured on an MRS solid plate to count the adhered bacteria (Nami et al., 2014; Popović et al., 2019).

The adhesion (%) = [(CFU/mL) adhered bacteria/(CFU/mL) added bacteria] × 100.

### Antibiotic Susceptibility

VITEK 2 Compact with AST-GP67 (REF 22226, bioMererieux, France) was used to access the antimicrobial susceptibility of the A8-1 to 15 clinical antibiotics, which included penicillin (PEN, 0.125–64 μg/mL), ampicillin (AMP, 0.5–32 μg/mL), high-level gentamicin (synergistic) (HLG, 500 μg/mL; GEN, 8–64 μg/mL), high-level streptomycin (HLS, 1,000 μg/mL), ciprofloxacin (CIP, 1–4 μg/mL), levofloxacin (LVX, 0.25–8 μg/mL), moxifloxacin (MXF, 0.25–8 μg/mL), erythromycin (ERY, 0.25–2 μg/mL), clindamycin (CLI, 0.15–2 μg/mL), quinupristin/dalofopine (QDA, 0.25–2 μg/mL), linezolid (LZD, 0.15–2 μg/mL), vancomycin (VAN, 1–16 μg/mL), tetracycline (TET, 0.15–2 μg/mL), tigecycline (TGC, 0.25–1 μg/mL), and nitrofurantoin (NIT, 16–64 μg/mL). According to the MIC obtained, the results were judged according to Clinical Laboratory Standard Institute criteria (CLSI M100 S28) (Clinical and Laboratory Standards Institute, 2018) and the European Food Safety Authority (EFSA) for assessment of bacterial resistance to antimicrobials (EFSA Panel on Additives and Products or Substances used in Animal Feed [FEEDAP], 2012).

### Hemolysis and Gelatinase Activity

Hemolytic activity of A8-1 was evaluated as described previously (Maturana et al., 2017). A8-1 was inoculated on Columbia agar supplemented with 5% (v/v) sheep blood and cultured at 37°C for 48 h. The hemolysis of single colonies on the plate was observed. Hemolytic activity can be divided into α-hemolysis, β-hemolysis, and γ-hemolysis.

Overnight cultured A8-1 was inoculated into gelatin medium (120 g/L gelatin, 5 g/L peptone, and 3 g/L beef extract, pH 6.8 ± 0.2) and incubated at 37°C for 48 h. Then, the tube was placed at 4°C for 1 h and it was observed whether there is liquefaction immediately. If the bacteria could produce gelatinase, there was liquid in the tube.

### Quantitative Assessment of Biofilm Formation

Quantitative assessment of biofilm formation was evaluated as shown previously (Zhang et al., 2016). Overnight bacterial cultures were washed with PBS twice and adjusted to OD_600_ = 1.0. The 50 μL of bacterial suspension was added to 150 μL of fresh MRS broth and incubated in 96-well plates at 37°C for 24 h. Also, 200 μL of MRS broth without bacteria was set as negative control. After 24 h, the culture medium was poured out. The wells were washed with sterile PBS three times to remove free-floating planktonic bacteria and then dried at room temperature. The biofilm was then fixed with methanol and stained with crystal violet. The control hole was rinsed with sterile water three times until it turned colorless. Then, 200 μL ethanol was added into each well, and optical density of stained adherent cells was measured at 595 nm by microplate reader. According to the cutoff OD (ODC), the biofilm-producing ability was determined as follows: OD ≤ ODC set as non-biofilm-producer (0), ODC < OD ≤ 2ODC set as weak biofilm producer (+), 2ODC < OD ≤ 4ODC set as moderate biofilm producer (++), and OD > 4ODC set as strong biofilm producer (+++).

### Virulence Gene and Virulence Activity Assay

The presence of virulence genes of A8-1 were detected by PCR, which included *gelE* (gelatinase), *cylA* (cytolysin), *hyl* (hyaluronidase), *asa1*/*agg* (aggregation substance), *esp* (enterococcal surface protein), *efaA* (endocarditis antigen), and *ace/acm* (collagen adhesion) (Wang et al., 2020). The primers and PCR conditions are listed in Supplementary Table 1.

For the virulence activity assay, tests in wax moth (*Galleria mellonella*) larvae were arranged (Martino et al., 2018). The *G. mellonella* larvae weighing about 300 mg (purchased from Tianjin Huiyude Biotech Company, Tianjin, China) were maintained on woodchips in the dark at 15°C until being used. The overnight cultures of *E. durans* A8-1, *E. faecalis* ATCC29212, and *B. infantis* CICC6069 suspension were adjusted with concentrations of 1 × 10^6^, 1 × 10^7^, and 1 × 10^8^ CFU/mL. Ten randomly selected larvae were used in each group. Each larva was inoculated the bacterial suspension *via* the rear left proleg using a 10 μL Hamilton animal syringe. The MRS medium was injected into the larvae and set as negative control. The treated *G. mellonella* larvae incubated at 37°C for 3 days, and the survival rate of the *G. mellonella* were recorded every 12 h.

### Impact of A8-1 on the Caco-2 Cell Membrane Integrity

Caco-2 cells were seeded into 24-well plates (1 × 10^5^ cells per well) and cultivated to a single layer. For the treatment, there were three treatment groups, S-A8-1 (competition group), A8-1 + S (exclusion group), and S + A8-1 (replacement group). S-A8-1, 1 × 10^7^ CFU/mL *S. typhimurium* ATCC14028 and 1 × 10^7^ CFU/mL A8-1 culture were added into the cell wells at the same time and incubated for 2 h; A8-1 + S, 1 × 10^7^ CFU/mL A8-1 culture were added into the cell wells and incubated for 2 h and then 1 × 10^7^ CFU/mL *S. typhimurium* ATCC14028 was added and incubated for another 2 h; S + A8-1, 1 × 10^7^ CFU/mL *S. typhimurium* ATCC14028 culture was added into the cell wells and incubated for 2 h and then 1 × 10^7^ CFU/mL A8-1 added and incubated for another 2 h. Finally, 1 × 10^7^ CFU/mL *S. typhimurium* ATCC14028 incubating solely with Caco-2 cells for 2 h was set as control. After incubation, the cell wells were washed three times by PBS to remove the unadhered *S. typhimurium* ATCC14028 cells. The amount of adhered *S. typhimurium* ATCC14028 to the Caco-2 cells were counted by bismuth sulfite agar plate (Popović et al., 2018; Kouhi et al., 2021). The inhibition rate of *S. typhimurium* adhesion to Caco-2 was calculated as:

Inhibition rate (%) = the counted adhered *S. typhimurium* ATCC14028 in treatment group (CFU/mL)/the counted adhered *S. typhimurium* ATCC14028 in control (CFU/mL).

Caco-2 cells were cultured and treated as mentioned into four treatment groups (*S. typhimurium* ATCC14028, A8-1, A8-1 + S, S + A8-1) and one control. After incubation, the supernatant of cell culture was collected after centrifuging at 1,500 rpm for 10 min at 4°C. The activity of extracellular alkaline phosphatase (AKPase) was assayed in the collected supernatant using a kit (Nanjing Jiancheng Technology Co., Ltd., Nanjing, China) as described (Lv et al., 2020). The AKPase unit was defined as 1 mg of phenol produced by 100 mL of cell culture supernatant reacted with the substrate at 37°C for 15 min. Cells treated with the same amount of sterile water were used as negative control. The release of lactate dehydrogenase (LDH) into the culture medium through damaged membranes was measured spectrophotometrically using a LDH Cytotoxicity Assay Kit Nanjing Jiancheng Technology Co., Ltd., Nanjing, China) according to the manufacturer’s protocol (García-Cayuela et al., 2014).

### Anti-inflammation Study Using Caco-2 Cells

#### Measurement of Cell Viability by 3-(4,5-Dimethylthiazol-2-yl)-2,5-Diphenyltetrazolium Bromide

It was carried out as described previously by Carasi et al. (2017). An 3-(4,5-Dimethylthiazol-2-yl)-2,5-diphenyltetrazolium bromide (MTT) colorimetric assay was used to monitor cell viability. Briefly, Caco-2 cells were seeded into 96-well plates (1 × 10^5^ cells per well). After treatment, cells were washed twice with PBS and incubated with 5 mg/mL MTT working solution for 4 h at 37°C. Then, the supernatant was removed, and the culture was resuspended in 150 μL of DMSO to dissolve MTT formazan crystals, followed by mixing on a shaker for 15 min. The absorbance was measured at 570 nm using a microplate reader. The effect of A8-1 culture and Lipopolysaccharides (LPS) on cell viability was assessed as the percentage of viable cells in each treatment group relative to untreated control cells, which were arbitrarily assigned a viability of 100%.

#### Measurement of Cell Cytokines by Enzyme-Linked Immunosorbent Assay and q-PCR

Quantification of cytokine levels in cell culture supernatants was determined by enzyme-linked immunosorbent assay (ELISA) and quantitative PCR (qPCR) (Mansour et al., 2014). Caco-2 cells were added into a 24-well cell culture plate according to 1 × 10^5^ cell/well and incubated until the cells grew to monolayer. There were two treatment groups, A8-1 + LPS and LPS + A8-1. For the A8-1 + LPS group, 2.5 × 10^6^ CFU A8-1 cells were added into Caco-2 cells and incubated for 6 h; the wells were washed three times by PBS, and 1 mL fresh DMEM was supplied and then 10 μg LPS was added and incubated for another 6 h. For the LPS + A8-1 group, 10 μg LPS was added into Caco-2 cells and incubated for 6 h; the wells were washed three times by PBS, and 1 mL fresh DMEM was supplied and then 2.5 × 10^6^ CFU A8-1 cells were added and incubated for another 6 h. The different treated Caco-2 cells and cell culture supernatant were collected at 6 and 12 h, separately. The contents of IL-8, IL-10, and TNF-α in the supernatant were detected by an ELISA kit (Sigma-Aldrich). Cell RNA extraction and relative mRNA expression of IL-8, IL-10, and TNF-α were determined according to the instructions of corresponding kits (Sigma-Aldrich). GAPDH was selected as the internal reference gene, and the relative mRNA expression levels of IL-8, IL-10, and TNF-α were calculated according to the 2^–Δ^ ^Δ^ *^CT^* method. The untreated Caco-2 cells were used as control, and all tests were performed in triplicate.

### Whole-Genome Sequence of A8-1

Whole-genome DNA of A8-1 was extracted by a kit (Applied Biosystems^®^ 4413021). The DNA concentration and purity was quantified with the NanoDrop2000. It was sequenced on the Illumina HiSeq™2000 platform at Gene *de novo* Biotechnology Co., Ltd. (Guangzhou, China). The reads were *de novo* assembled by SOAPdenovo version 2.04, Li et al. (2009) and the genome sequence was improved by GapCloser. For the analysis of specific gene sequences, such as virulence genes, heavy metal resistance genes, antibiotic resistance genes, and efflux gene sequences in the bacterial genome, the genomes were analyzed and retrieved in the BIGSdb.^1^ The predicted genes of *E. durans* A8-1 were compared with the comprehensive antibiotic resistance database (CARD)^2^ (McArthur et al., 2013) and virulence factors database (VFDB)^3^ (Chen et al., 2016) for identifying antibiotic resistance and virulence factors. Furthermore, the ResFinder 3.0^4^ (Zankari et al., 2012) and PathogenFinder 1.1^5^ (Cosentino et al., 2013) were used for identifying the acquired antibiotic resistance genes and pathogenicity factors, respectively. Clustered regularly interspersed short palindromic repeats (CRISPR) and prophage sequences were identified by CRISPR Finder^6^ (Grissa et al., 2007). The genome sequences have been submitted to antiSMASH bacterial version^7^ to search for the secondary metabolite biosynthetic gene clusters.

### Statistical Analysis

The test and analysis of variance (ANOVA), STAMP10, GraphPad Prism 7, and SPSS V20.0 (IBM Inc., IL, United States) were used to perform statistical analyses. Data were presented as means ± SEM. *P* < 0.05 was considered significant differences.

## Results

### Isolation and Identification of A8-1

**FIGURE 1:**
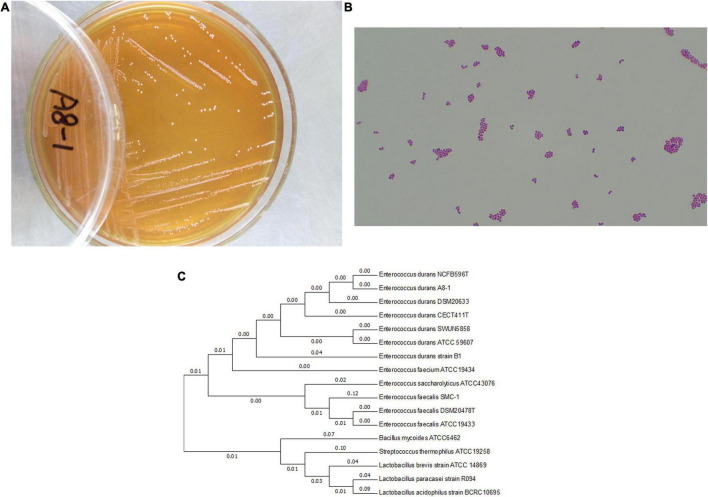
The growth of *Enterococcus durans* A8-1 on MRS plate **(A)**; the Gram staining observation at microscope **(B)** (Olympus CX23, 40×); and phylogenetic tree based on the 16S rRNA gene sequences **(C)**, which was inferred by using the maximum likelihood method and conducted in MEGA7.

The Gram-positive and cocci-shaped bacteria were selected from a fecal sample of healthy infants. The colony morphology had a sticky, translucent white and mucoid appearance on MRS agar. Except cell morphology, Gram staining (G^+^), catalase (negative) and oxidase production (negative), nitrate reduction test (negative), a final strain of A8-1 was confirmed by 16S rRNA sequence analysis (Figures 1A–C). The 16S rRNA was submitted to NCBI with the accession number of MH385353.

### Analysis of Probiotic Characteristics of *Enterococcus durans* A8-1

#### Acid and Bile Salt Tolerance Under Aerobic and Anaerobic Conditions

**FIGURE 2:**
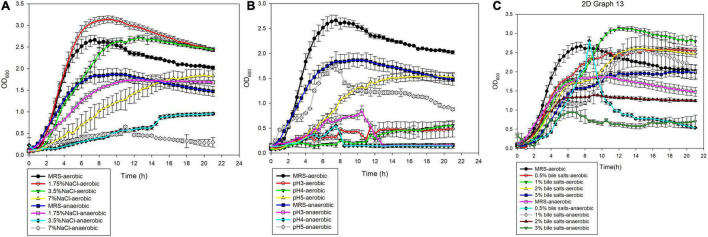
*In vitro* tolerance of *Enterococcus durans* A8-1 under aerobic and anaerobic conditions with **(A)** 1.75, 3.5, and 7% NaCl; **(B)** pH 3, 4, and 5; **(C)** 0.5, 1, 2, and 3% bile salts.

A8-1 had different tolerance in different environments in our experiment. Under aerobic conditions, A8-1 could survive at pH 5.0 in MRS medium, and the maximum cell density reached about 50% of the density in normal MRS under aerobic conditions (Figure 2). A8-1 showed great tolerance to bile salt and NaCl. It was found that the growth of A8-1 in 1.75 and 3.5% NaCl was better than that in MRS (ODmax: 2.841 vs. 2.322, *P* < 0.05; 2.619 vs. 2.322, *P* < 0.05). The maximum biomass of A8-1 in a 0.5, 1, and 2% bile salt environment were higher than that of the control group (Figure 2A). Under anaerobic conditions, the maximum OD_600_ of A8-1 was close to the control at pH 5.0 (Figure 2B), and the strain could grow well under 1% bile salt (ODmax: 2.497 vs. 1.713, *P* < 0.01) and 1.75% NaCl (ODmax: 1.739 vs. 1.713, *P* > 0.05) (Figure 2C). Under aerobic conditions, compared with the MRS control group, the lag phase of A8-1 was the shortest at 2% bile salts and 7% NaCl, was delayed under pH 5. Under anaerobic conditions, compared with MRS control, the lag phase of A8-1 was the shortest at 1% bile salt, and the lag phase of A8-1 was shortened with NaCl added (Table 1).

**TABLE 1:** Comparison of acid, NaCl, and bile salt tolerance of *Enterococcus durans* A8-1 cultured at aerobic and anaerobic conditions.

Medium	Aerobic			Anaerobic		
	ODmax	LSD (h)	*R* ^2^	ODmax	LSD (h)	*R* ^2^
MRS	2.322	1.5944	0.9266	1.713	0.6206	0.9375
1.75% NaCl	2.841	1.6536	0.948	1.739	0.3693	0.9947
3.5% NaCl	2.619	1.5034	0.9889	0.396	0.5392	0.7078
7% NaCl	1.974	1.1152	0.9982	1.418	0.2969	0.9764
pH 3	0.476	NA*	NA	0.5662	NA	NA
pH 4	1.513	1.1036	0.9363	0.209	NA	NA
pH 5	1.585	2.1805	0.9927	1.234	0.933	0.5311
0.5% Bile salts	2.539	1.0611	0.9956	1.104	NA	NA
1% Bile salts	3.018	1.496	0.9735	2.497	0.1764	0.9209
2% Bile salts	2.595	0.7329	0.9825	1.288	0.8741	0.9382
3% Bile salts	1.97	1.5058	0.9946	0.704	NA	NA

*NA, not fit for the Gompertz growth curve analysis.

#### *In vitro* Adherence Assay

Compared with *L. rhamnosus* GG BL379 (positive control), A8-1 showed higher adhesion to mucin (*P* < 0.01), BSA (*P* < 0.01), and collagen. The adhesion ability of A8-1 to mucin, collagen, and BSA was 5.2, 1.6, and 5.6 times higher than *L. rhamnosus* GG BL379. For the adhesion to Caco-2 cells, the adhesion of A8-1 was 38.47%, which is higher than *L. rhamnosus* GG BL379 (38.47 vs. 11.7%, *P* < 0.05) determined in our study.

For the surface adhesion ability, A8-1 showed a high hydrophobicity of 79.2 ± 3.1% in chloroform and moderate hydrophobicity of 49.2 ± 4.4% in xylene; it had 51.7 ± 4.5% for the auto-aggregation after 5 h of incubation and was able to co-aggregate with *S. typhimurium* with a co-aggregation percentage of 66.2 ± 2.9%.

#### Antibacterial Activity Analysis

The fermentation supernatant of A8-1 showed different antibacterial activity against the indicator strains for *P. aeruginosa* PA01 and *E. coli* ATCC35218, the MIC was 25 μL supernatant, and for the *S. aureus* ATCC25923, *P. aeruginosa* ATCC27853, *E. hormaechei* ATCC700323, *S. typhimurium* ATCC14028, the MIC was 50 μL supernatant.

### Protect Effect to the Caco-2 Cell

#### Protection Effect of A8-1 to the Caco-2 Cell Membrane Integrity

**FIGURE 3:**
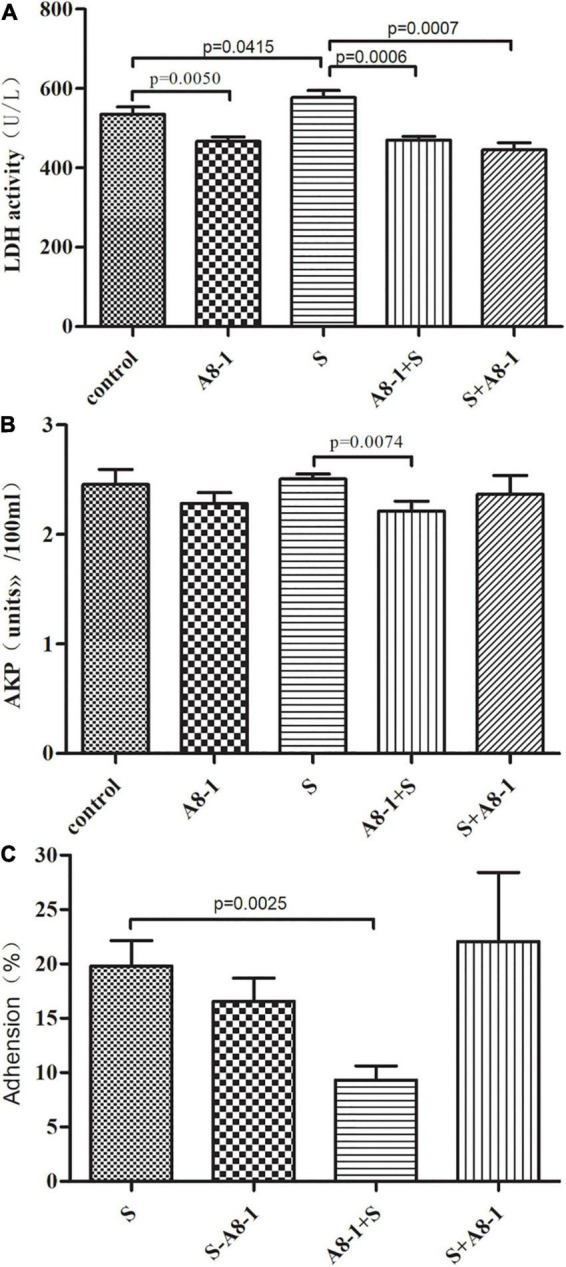
Protective effect of *Enterococcus durans* A8-1 to the Caco-2 cell membrane integrity by lactate dehydrogenase (LDH) **(A)** and AKP **(B)** activity assay and the adhesion rate of A8-1 to the Caco-2 cell **(C)**.

The enzyme activities of LDH and AKP in the supernatant of cell culture were selected as indicators for the integrity of the Caco-2 cell membrane (Figures 3A,B). Compared with the control group, it was found that the LDH activity in the A8-1 group was significantly reduced compared with the control group (467.20 vs. 535.58, *P* = 0.005) and was increased in the *S. typhimurium* ATCC14028 group (577.92 vs. 535.58, *P* = 0.042), whereas AKP activity was changed with a similar trend to LDH but no statistical difference. In the A8-1 + S group, activities of LDH and AKP in the Caco-2 cell supernatant were all significantly decreased compared with that of the Salmonella group (470.46 vs. 577.92, *P* < 0.001; 2.21 vs. 2.51, *P* = 0.007). Even in the S + A8-1 group, LDH activity was still significantly lower than that of the Salmonella group (445.61 vs. 577.92, *P* < 0.001). Those results show that strain A8-1 could protect the integrity of the Caco-2 cell membrane in pretreatment and inhibit the damage of *Salmonella* to Caco-2 cells.

#### A8-1 Competitively Inhibited the Adhesion of *Salmonella typhimurium* to Caco-2 Cell

The plate counting method was used to explore the antagonism of A8-1 against the adhesion of *S. typhimurium* ATCC14028 to Caco-2 cells. It is found that, in S-A8-1 (competition group), A8-1 can reduce the adhesion of *S. typhimurium* to cells without statistical difference (19.8 vs. 16.5%, *P* > 0.05). In A8-1 + S (exclusion group), the adhesion of *S. typhimurium* to Caco-2 cells was significantly reduced (19.8 vs. 10.5%, *P* = 0.002) possibly because A8-1 could inhibit the growth of *S. typhimurium* and occupied the binding sites on the surface of the Caco-2 cells. However, there was no statistical difference for the adhesion to Caco-2 cells between S + A8-1 (replacement group) and the *S. typhimurium* group (19.8 vs. 22.1%, *P* = 0.592) (Figure 3C). Those results showed that A8-1 competitively inhibited the adhesion of *S. typhimurium* to Caco-2 cell.

#### A8-1 Reduced IL-8 and Increased IL-10, TNF-α Secretion in Response to LPS Stimulation in Caco-2 Cells

**FIGURE 4:**
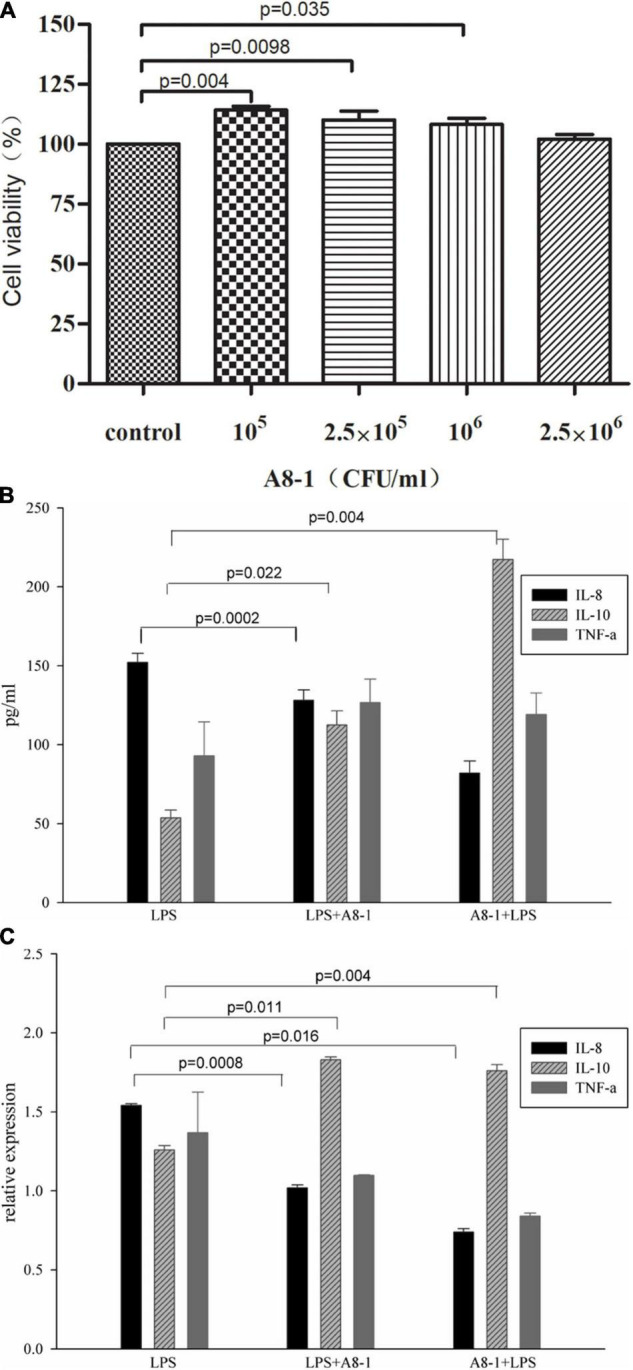
The Caco-2 cell viability with incubation of 10^5^, 2.5 × 10^5^, 10^6^, and 2.5 × 10^6^CFU/mL of A8-1 **(A)**. Then, 10 μg/mL LPS was added to the medium to induce the inflammation of Caco-2 cells, the anti-inflammation ability of 2.5 × 10^6^CFU/mL A8-1 was detected by IL-8, IL-10, and TNF-α through ELISA **(B)** and qPCR **(C)**.

Results of cell viability treated by A8-1 are shown in Figure 4A. Different concentrations of A8-1 cells with 1 × 10^5^, 2.5 × 10^5^, and 1 × 10^6^ CFU/mL could all significantly increase the Caco-2 cell viability (114.18 vs. 100%, *P* = 0.004; 110.04 vs. 100%, *P* = 0.0098; 108.14 vs. 100%, *P* = 0.035) except the 2.5 × 10^6^ CFU/mL group (102.10 vs. 100%, *P* = 0.140). So 2.5 × 10^6^ CFU/mL of A8-1 cells was selected for the following assays.

The 10 μg/mL LPS was added to the medium and induced the inflammation of Caco-2 cells, and the anti-inflammation ability of A8-1 was detected by IL-8, IL-10, and TNF-α through ELISA and qPCR (Figures 4B,C). Compared with the LPS treatment cells, IL-8 were decreased in both A8-1 + LPS and LPS + A8-1 groups and showed significant difference between LPS + A8-1 and LPS groups (82.11 vs. 152.23 pg/mL, *P* = 0.002); the IL-10 were significantly increased in both A8-1 + LPS and LPS + A8-1 groups (217.3 vs. 53.7 pg/mL, *P* = 0.004; 112.5 vs. 53.7 pg/mL, *P* = 0.022). TNF-α increased in both intervention groups, but there was no significant difference compared with LPS group (126.7 vs. 93.0 pg/mL, *P* = 0.265; 119.1 vs. 93.0 pg/mL, *P* = 0.362). For the relative expression of mRNA, the IL-8 in A8-1 + LPS and LPS + A8-1 groups were significantly decreased (1.54 vs. 1.02, *P* = 0.0016; 1.54 vs. 0.74, *P* = 0.0008); IL-10 were significantly increased in both A8-1 + LPS and LPS + A8-1 groups (1.83 vs. 1.26, *P* = 0.004; 1.76 vs. 1.26, *P* = 0.011). TNF-α expression was decreased in both groups without statistical difference (1.36 vs. 1.09, *P* = 0.4059; 1.36 vs. 0.84, *P* = 0.1784). Those results showed that A8-1 could reduce the secretion of IL-8 and increase the secretion of IL-10 and TNF-α in response to LPS stimulation in Caco-2 cells.

### Safety Evaluation of A8-1

Susceptibility of *E. durans* A8-1 to antibiotics was determined by measuring MICs, and the results were compared to the cutoff values for *Enterococcus* species as defined by EFSA and CLSI. *E. durans* A8-1 was found to be resistant only to clindamycin but was susceptible to penicillin, ampicillin, high-level gentamicin (synergistic), high-level streptomycin, ciprofloxacin, levofloxacin, moxifloxacin, erythromycin, quinupristin/dalofopine, linezolid, vancomycin, tetracycline, tigecycline, and nitrofurantoin (Table 2). A8-1 showed no hemolytic activity (Supplementary Figure 1A) and no gelatin hydrolysis activity (Supplementary Figure 1B). It was identified as a weak biofilm producer (++). In the nine tested virulence related genes, there showed only *asa1* gene positive in A8-1.

**TABLE 2:** Minimum inhibitory concentrations (MICs) of *Enterococcus durans* A8-1 against 15 antimicrobials, and the antimicrobial susceptibility was evaluated by CLSI-2018 and FEEDAP-EFSA-2012.

Antimicrobials	MIC	Cutoff values		Antimicrobial susceptibility
		CLSI	FEEDAP-EFSA	
Penicillin	2	8	4	S
Ampicillin	≤2	8	4	S
Ciprofloxacin	≤0.5	1	4	S
Levofloxacin	0.25	2	NA	S
Moxifloxacin	≤0.25	0.5	NA	S
Erythromycin	≤0.25	0.5	4	S
Clindamycin	≥8	8	8	R*
Quinupristin/dalofopine	1	4	NA	S
Linezolid	2	4	NA	S
Vancomycin	≤0.5	4	4	S
Tetracycline	≤1	4	2	S
Tigecycline	≤0.12	0.2	NA	S
Nitrofurantoin	32	64	NA	S
high-level gentamicin (synergistic)				SYN-S
high-level streptomycin				SYN-S

*For Enterococcus spp., clindamycin may appear active in vitro but is not effective clinically and should not be reported as susceptible as described in CLSI.

*NA, Not available*

**FIGURE 5:**
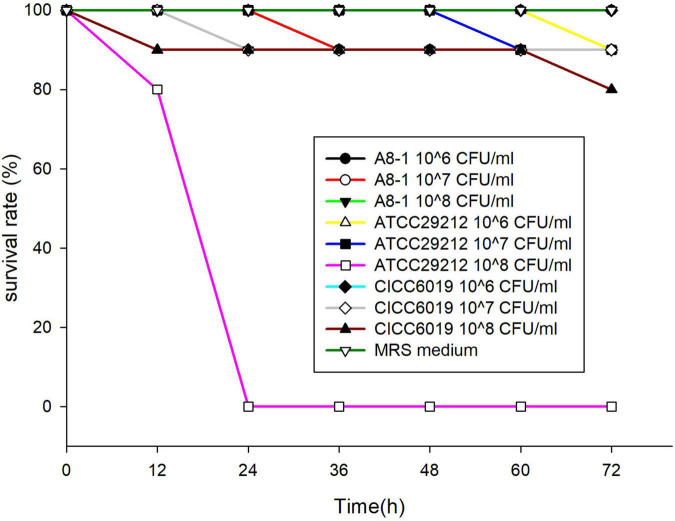
Survival curves of *Galleria mellonella* larvae were recorded for 72 h after injection with 10 μL of *Enterococcus durans* A8-1, *Enterococcus faecalis* ATCC29212, and *Bifidobacterium infantis* CICC6096 at concentrations of 1 × 10^6^, 1 × 10^7^, and 1 × 10^8^ CFU/mL, respectively, and the 10 μL of MRS medium was used as negative controls.

For the virulence assay with *G. mellonella* larvae, after 72 h injection, except the 10^8^CFU/mL group of *E. faecalis* ATCC29212, all larvae had a 90% survival rate in both the 10^7^ and 10^8^CFU/mL groups. All larvae survived in the control and the 10^6^CFU/mL groups. The survival curves were analyzed statistically using Log-rank (Mantel-Cox) Test, and there were no statistical differences in *E. durans* A8-1 and *B. infantis* CICC6096 within 10^7^ and 10^8^CFU/mL groups (*P* > 0.05). Apparently, A8-1 could be considered safe (Figure 5).

### Whole-Genome Sequence of A8-1

The circular chromosome of *E. durans* A8-1 contains 2,877,218 bp, 37.92% GC-content, and 56 tRNA genes (Table 3). Genome annotation at the RAST server showed that the C-1 genome encodes 2,752 proteins, and the corresponding functional categorization by COG annotation is in Supplementary Table 2. The sequence data of the *E. durans* A8-1 genome were deposited into NCBI and can be accessed *via* accession number PRJNA769572. The GO function annotation map of genome is shown in Supplementary Table 3. There were 1,123 genes related to biological process (BP), 590 genes related to cell composition (CC), and 757 genes related to molecular function (MF). Among the genes involved in BP, there were two bio-adhesion–related genes (A8-1_0276, Zinc-binding lipoprotein *adcA*; A8-1_2290, Metal ABC transporter substrate-binding lipoprotein), 85 cell colonization–related genes, 268 binding ability genes, and four antioxidant genes (A8-1_0078, Glutathione peroxidase; A8-1_2302, Manganese catalase; A8-1_2334, carboxymuconolactone decarboxylase; A8-1_2613, peroxiredoxin).

**TABLE 3:** Genomic analysis of A8-1.

Characteristic	Number	Characteristic	Number
Genome size (bp)	2,877,218	CRISPR	2
Scaffolds	102	tRNA	56
GC content (%)	37.92	Transposon PSI	17
Code genes	2,752	GIs	15

The probiotic-related genes in the genome were also analyzed. The cholylglycine hydrolase (EC 3.5.1.24) gene responsible for bile salt hydrolysis action was identified in one copy within the A8-1 genome (A8-1_2053). Fibronectin/fibrinogen-binding protein (A8-1_2247) and collagen-binding protein (A8-1_0314) were found in the genome allowing them to bind the GI tract, suggesting an important role in adhesion and colonization in intestinal mucosal surfaces. Also, the resistance to hydrogen peroxide is imparted by genes alkyl hydroperoxide reductase (*ahp*, A8-1_2612, A8-1_2613) and NADH peroxidase (npr, A8-1_1888, A8-1_2186, and A8-1_2466) that were found in the genome. For the polysaccharide biosynthesis–related genes, there were eight genes located in the genome, including A8-1_ 0155 (polysaccharide biosynthesis glycosyltransferase), A8-1_0235 (polysaccharide core biosynthesis protein RfaS), A8-1_0854 (polysaccharide transport system ATP-binding protein), A8-1_1612 (sugar transferase), A8-1_1617 (polysaccharide cholinephosphotransferase), A8-1_1621 (polysaccharide core biosynthesis protein RfaS), A8-1_1639 (polysaccharide chain length determining protein CapA), and A8-1_1764 (polysaccharide biosynthesis protein). In addition, based on the previous studies, we also screened for a set of genes involved in imparting important probiotic functions as described in Table 3.

The drug-resistance and virulence genes were annotated by database of CARD and VFDB. Two aminoglycoside resistance–related genes *AAC (6′)-IIH* and *AAC (6′)-IID*; three β-lactam resistance genes *mecC*, *mecB*, and *mecA*; and one fluoroquinolone resistance gene *mfd* were predicted in CARD. Regarding the possibility of acquired resistance by horizontal gene transfer (HGT), there was no detection of any acquired antibiotic resistance genes. In addition, *efaA/scbA* was found in virulence gene prediction (A8-1_2290, endocarditis specific antigen); however, the similarity score was only 50.1%. Meanwhile, the *asa1* gene detected by PCR was not found in the genome sequencing results, which may explain the non-toxic activity to the *G. mellonella* larvae. In addition to the above genes, there predicated some genes responsible for the secondary metabolites, such as *bsh* (A8-1_2053, bile salt hydrolase), which may be related to the bile salt tolerance and survival in the intestinal tract for A8-1; *cap8E* (A8-1_1630, capsular polysaccharide synthesis enzyme), *cap8E* (A8-1_1631, capsular polysaccharide synthesis enzyme), *cas4J* (A8-1_1631, capsular polysaccharide biosynthesis protein) genes related to capsular polysaccharide synthesis were also found in the genome, which may contribute to protect bacteria itself and resist the phagocytosis of host cells; *fliN* (A8-1_1957, flagella motor switch protein), which is involved in synthesis of flagellin, which could be recognized by Toll like receptor *TLR5*, and activate innate immunity, upregulate the expression of tight junction protein Occludin and mucin and protect the intestinal barrier.

## Discussion

Probiotics, the microorganisms referred to are non-pathogenic bacteria and are considered “friendly germs” due to the benefits they offer to the gastrointestinal tract and immune system. Probiotic *Enterococcus* spp. are mainly from the gut of human and animal and can be detected in fecal samples, which are more competitive than isolates from other environments and deserve more attention for probiotic screening (Rodríguez et al., 2015; Zheng et al., 2015). Except the origin host, in the screening for new probiotic strains, probiotic characteristics of the isolates should be analyzed, including stress tolerance, adhesion, antibacterial ability, anti-inflammatory ability, antibiotic resistance, and toxicity (AlKalbani et al., 2019). Our study aimed to assess the potential probiotic properties and safety of an *E. durans* strain A8-1 isolated from feces of a healthy Chinese infant.

In our results, A8-1 grew faster and showed higher antistress ability. By simulating the gastrointestinal environment, it was found that, under the aerobic conditions of pH 5.0, 3% bile salt, and 7% NaCl, the growth of *E. durans* A8-1 could reach more than 50% of the control group, and this indicated that A8-1 has the potential to pass through the intestinal contents and reach the intestinal colonization site. Bile salt tolerance has generally been considered more important during probiotic selection than that of other properties, such as gastric and pancreatic tolerance (Masco et al., 2007). The growth of the strain was stimulated under bile salt and NaCl, which suggested that we can optimize the fermentation conditions to promote better growth and faster enrichment of bacteria cells. In the genome of A8-1, the bile salt hydrolase–related gene *bsh* was found, and the presence of bile salt hydrolase in probiotics renders them more tolerant to bile salts (Hussein et al., 2020), which may interpret the better tolerance of A8-1 to higher concentration of bile salt.

The first step for good probiotics to exert probiotics in the host is to adhere to the cell surface, which is also the basis for probiotics to show the barrier protection function. Also, for probiotic enterococci, it is an important factor in colonization and competitive exclusion of enteropathogens (Nueno-Palop and Narbad, 2011). The stronger the adhesion ability of the bacteria, the higher the probability of colonization and survival in the intestinal tract. Compared with *L. rhamnosus* BL379, A8-1 had higher adhesion ability to the tested proteins of mucin, BSA, and collagen. The human intestinal epithelial-like Caco-2 cell line is often used as an intestinal epithelial cell model (Jose et al., 2017). The adhesion rate of A8-1 to Caco-2 cells was 30%, and this result showed that the adhesion performance of the A8-1 to various proteins and cells was different. Also, the considerably high level of hydrophobicity, auto-aggregation, and co-aggregation of A8-1 could enable the bacterial cell to adhere to host epithelial cells and allow the formation of a barrier to prevent the colonization of pathogens on surfaces of the mucosa (Nami et al., 2020; Fonseca et al., 2021). Similarly, whether the strain has the same adhesion ability *in vivo* and *in vitro* also needs to be considered (Banwo et al., 2013). Metabolites produced by probiotics have antibacterial activities, such as organic acids, hydrogen peroxide, and bacteriocin (İspirli et al., 2015). A8-1 has a better antibacterial effect on G^–^ than G^+^, which may be related to the similar bacterial structure of A8-1 (G^+^) and two G + indicator bacteria (*E. faecalis* ATCC 29212 and *S. aureus* ATCC 5923). Moreover, bacteriocin showed a narrow antibacterial spectrum against the same related strains. In this study, the growth of the indicator bacteria was used to determine the antibacterial ability of *Enterococcus* isolates, but the specific types and production of antibacterial active substances were not discussed. To further analyze the bacteriostatic mechanism of A8-1, the eight specific polysaccharide biosynthesis–related genes, which were annotated in the genome sequence of A8-1, deserve more attention.

Lipopolysaccharide is often used as a substance to induce an inflammatory reaction, which can make cells produce inflammation and stimulate the expression of inflammatory cytokines (Drakes et al., 2004). Our results show that the inflammatory response induced by LPS could be alleviated and IL-8 mRNA could be reduced after being pretreated with A8-1. The expression of TNF-α mRNA was decreased, but there was no significant change in the supernatant of each treatment group (*P* > 0.05). A8-1 may have the potential to inhibit inflammatory response. When inflammation occurs, A8-1 can reduce the expression of cytoinflammatory factors to reduce the inflammatory response of cells to LPS. It is worth noting that probiotics have highly diverse effects on the level of immune regulatory cytokines, mainly related to the specificity of strains and cell lines (Kook et al., 2019).

*Enterococcus*, as the original symbiotic bacteria in the intestine, has dual characteristics of probiotic function and potential pathogenicity. Therefore, it is necessary to compare and analyze the functional characteristics and safety at the strain level (Klimko et al., 2020). To evaluate the safety of A8-1, susceptibility to antibiotics, hemolytic activity, gelatin hydrolysis activity, virulence-related genes, and the virulence assay were all carried out. In the antibiotic susceptibility test, A8-1 was only resistant to clindamycin, which may be related to the inherent resistance of enterococci, which was verified in *E. durans* KLDS 6.0930 (Li et al., 2018). Antibiotics linezolid and vancomycin are often used as the last resort for G^+^ pathogen infection. β-lactams (penicillin, ampicillin), and aminoglycoside antibiotics are generally the preferred drugs for the treatment of *enterococci* infection, which may make the strains more resistant to such antibiotics (Tsai et al., 2012). A8-1 was sensitive to the above antibiotics. Bacterial toxicity should be evaluated by phenotype and genotype. The *esp* gene may be involved in the formation of biofilm, but it is not the only factor determining the producing of biofilm (Fallah et al., 2017; Kook et al., 2019). No *esp* gene was detected in A8-1. The ability of producing biofilm was considerate lower, which may be related to the lack of *esp* gene. A8-1 was *gleE* gene negative with no gelatin hydrolase (Popović et al., 2018). Furthermore, there were no obvious pathogenicity or virulence genes found in A8-1. This correlates with the observed phenotype in the *G. mellonella* model due to the low mortality rates that were obtained, which is similar behavior to the larvae inoculated with *L. lactis* strains reported by Martino (Martino et al., 2018).

The whole-genome sequencing of bacteria is a convenient way to determine antibiotic-resistant genotypes and predict the corresponding resistance phenotypes, and the phenotype does not always completely reflect the genotype. Through comparative analysis of ARDB, it predicted aminoglycosides, β-lactam antibiotics, and fluoroquinolone-related genes in A8-1, but A8-1 had no corresponding resistance phenotype. It may be because the expression of the drug-resistant gene was silenced or the transcription process was not completed, resulting in the absence of resistance phenotype. A8-1 is resistant to clindamycin, which is usually an inherent antibiotic resistant to *Enterococcus*, and the predicted *lasA*, *lmrB*, *lmrC*, and *lmrD* genes contributed to the clindamycin-resistance through the efflux pump function (Hollenbeck and Rice, 2012). However, antibiotic resistance may have a positive effect on probiotics. For some antibiotic-resistant *enterococci*, it can effectively maintain the natural balance of intestinal flora in the process treatment (Strompfová et al., 2004).

In the current research, we only explored the impact of potential probiotic A8-1 on intestinal epithelial cell membranes and the regulation of cell inflammation. In the follow-up experiments, the transepithelial electrical resistance (TER) should be measured, and the cell tight junction protein can be further detected to explain the effect of A8-1 on the maintenance of gut permeability and intestinal barrier function and interpret the mechanism of inflammation suppression through the inflammatory response-related signal pathways. Furthermore, animal models will be established to evaluate the safety and functionality of *E. durans* A8-1 before potential use in applications.

## Conclusion

In summary, we have identified a strain of *E. durans* that is able to tolerate and survive the simulated gastric and intestinal juices and has the potential to colonize the intestinal epithelial cells. Furthermore, we also showed that it contains no obvious pathogenicity or virulence genes. Taken together, our findings suggest the efficacy of probiotic *E. durans* A8-1 in exerting an adherence to the cell surface to show the barrier protection function and competitive exclusion of enteropathogens with reductions in the levels of inflammatory cytokines. According to the results of these evaluated attributes, *E. durans* strain A8-1 could be a promising probiotic candidate for applications.

## Data Availability Statement

The datasets presented in this study can be found in online repositories. The names of the repository/repositories and accession number(s) can be found in the article/Supplementary Material.

## Author Contributions

BH and YC: conceptualization and writing—review and editing. YZ, JW, and LS: main experiments. LL and JY: genome analysis. RD: data and bacterial curation. BH and YZ: writing—original draft preparation. BH: supervision. BH and RD: funding acquisition. All authors agreed to be accountable for the content of the work.

## Conflict of Interest

The authors declare that the research was conducted in the absence of any commercial or financial relationships that could be construed as a potential conflict of interest.

## Publisher’s Note

All claims expressed in this article are solely those of the authors and do not necessarily represent those of their affiliated organizations, or those of the publisher, the editors and the reviewers. Any product that may be evaluated in this article, or claim that may be made by its manufacturer, is not guaranteed or endorsed by the publisher.

## Footnotes

1
https://bigsdb.pasteur.fr/

2
http://arpcard.mcmaster.ca/

3
http://www.mgc.ac.cn/VFs/main.htm

4
https://cge.cbs.dtu.dk//services/ResFinder/

5
https://cge.cbs.dtu.dk/services/PathogenFinder/

6
http://crispr.i2bc.paris-saclay.fr/Server/

7
https://antismash.secondarymetabolites.org/
