# Supplementary material for: Probiotic Potential Analysis and Safety Evaluation of Enterococcus durans A8-1 Isolated From a Healthy Chinese Infant
Source: Front Microbiol. 2021 Dec 14;12:799173. doi: 10.3389/fmicb.2021.799173 (PMC8712863; doi:10.3389/fmicb.2021.799173)
Supplement: Supplementary file 1 [file Data_Sheet_1.docx]

Figure S1 The hemolytic activityon blood agar plate (A)and gelatin hydrolysis activity (B) detected in gelatin mediumof *Enterococcus durans* A8-1.There were no red blood cells lysis means no hemolysin production; In the gelatin medium, the gelatinase producing *Enterococcus faecalis* C66 could hydrolyze the gelatin, and the *Enterococcus durans* A8-1 had no gelatinase activity, the gelatin medium tubes were in solid in A8-1 and blank.


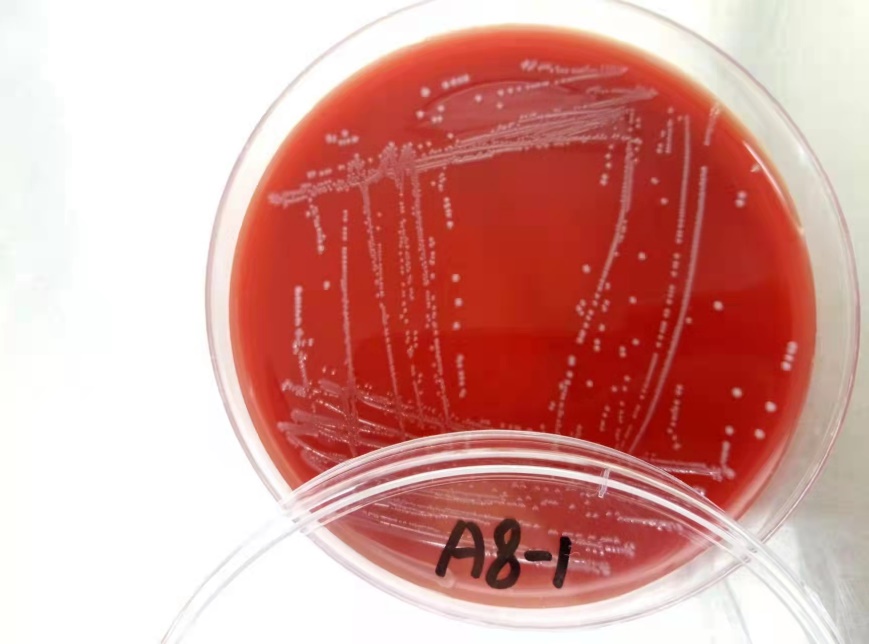
A


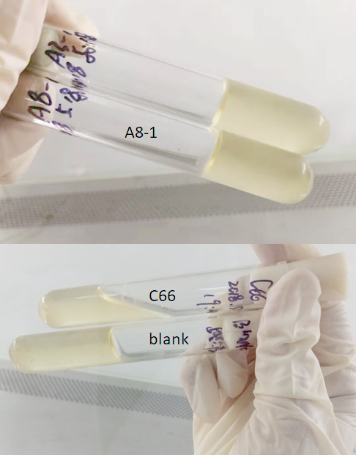
B

Table S1 The primers and PCR conditions for the virulence genes detection

| gene | Primers | 5’-3’ | Amplified fragment（bp） | Annealing temperature（℃） |
| --- | --- | --- | --- | --- |
| *asa1* | *asa1*-F | GCACGCTATTACGAACTATGA | 375 | 56 |
|  | *asa1*-R | TAAGAAAGAACATCACCACGA |  |  |
| *agg* | *agg*-F | AAGAAAAAGAAGTAGACCAAC | 1553 | 56 |
|  | *agg*-R | AAACGGCAAGACAAGTAAATA |  |  |
| *cylA* | *cylA*-F | ACTCGGGGATTGATAGGC | 688 | 54 |
|  | *cylA*-R | GCTGCTAAAGCTGCGCTT |  |  |
| *esp* | *esp*-F | AGATTTCATCTTTGATTCTTGG | 510 | 56 |
|  | *esp*-R | AATTGATTCTTTAGCATCTGG |  |  |
| *hyl* | *hyl*-F | ACAGAAGAGCTGCAGGAAATG | 276 | 58 |
|  | *hyl*-R | GACTGACGTCCAAGTTTCCAA |  |  |
| *efaAfm* | *efaAfm*-F | AACAGATCCGCATGAATA | 735 | 56 |
|  | *efaAfm*-R | CATTTCATCATCTGATAGTA |  |  |
| *gelE* | *gelE*-F | ACCCCGTATCATTGGTTT | 419 | 56 |
|  | *gelE*-R | ACGCATTGCTTTTCCATC |  |  |
| *acm* | *acm*-F | GGCCAGAAACGTAACCGATA | 135 | 54 |
|  | *acm*-R | AACCAGAAGCTGGCTTTGTC |  |  |
| *ace* | *ace*-F | AAAGTAGAATTAGATCCACAC | 320 | 54 |
|  | *ace*-R | TCTATCACATTCGGTTGCG |  |  |

Table S2 Cluster of Orthologous Groups summary of annotated proteins in*Enterococcus durans* A8-1 genome

| Code | Functional-Categories | Genenumber |
| --- | --- | --- |
| B | Chromatin structure and dynamics | 1 |
| C | Energy production and conversion | 91 |
| D | Cell cycle control, cell division, chromosome partitioning | 25 |
| E | Amino acid transport and metabolism | 204 |
| F | Nucleotide transport and metabolism | 81 |
| G | Carbohydrate transport and metabolism | 276 |
| H | Coenzyme transport and metabolism | 65 |
| I | Lipid transport and metabolism | 63 |
| J | Translation, ribosomal structure and biogenesis | 156 |
| K | Transcription | 224 |
| L | Replication, recombination and repair | 171 |
| M | Cell wall/membrane/envelope biogenesis | 139 |
| N | Cell motility | 13 |
| O | Posttranslational modification, protein turnover, chaperones | 65 |
| P | Inorganic ion transport and metabolism | 148 |
| Q | Secondary metabolites biosynthesis, transport and catabolism | 34 |
| R | General function prediction only | 319 |
| S | Function unknown | 238 |
| T | Signal transduction mechanisms | 111 |
| U | Intracellular trafficking, secretion, and vesicular transport | 30 |
| V | Defense mechanisms | 74 |

Table S3 Gene Ontology enrichment analysis in *Enterococcus durans* A8-1 genome

| **Ontology** | **Class** | **Gene Number** |
| --- | --- | --- |
| Biological Process | metabolic process | 354 |
|  | cellular process | 294 |
|  | biological adhesion | 2 |
|  | signaling | 4 |
|  | single-organism process | 247 |
|  | response to stimulus | 31 |
|  | localization | 85 |
|  | multi-organism process | 1 |
|  | biological regulation | 71 |
|  | cellular component organization or biogenesis | 32 |
|  | detoxification | 2 |
| Molecular Function | transcription factor activity, protein binding | 3 |
|  | nucleic acid binding transcription factor activity | 18 |
|  | catalytic activity | 374 |
|  | structural molecule activity | 32 |
|  | transporter activity | 57 |
|  | binding | 268 |
|  | antioxidant activity | 4 |
|  | molecular transducer activity | 1 |
| Cellular Component | cell | 163 |
|  | nucleoid | 1 |
|  | membrane | 85 |
|  | macromolecular complex | 70 |
|  | organelle | 17 |
|  | organelle part | 12 |
|  | membrane part | 79 |
|  | cell part | 163 |
